# Psychosis in Huntington's disease: A case supporting multidisciplinary and early palliative care

**DOI:** 10.1177/18796397261472525

**Published:** 2026-07-29

**Authors:** Vy Le, Edith Sigler, Jonathan Liu, Hiba Abualama, Kamalakar Surineni

**Affiliations:** 1 Department of Psychiatry, 2331 UNC School of Medicine, Chapel Hill, NC, USA; 2 Department of Internal Medicine, 21638 KU School of Medicine – Wichita, KS, USA; 3 Department of Psychiatry & Behavioral Sciences, 21638 KU School of Medicine – Wichita, KS, USA

**Keywords:** neuropsychiatry, psychiatry, neurophysiology, movement disorders, cognition

## Abstract

Following an abrupt medication switch from olanzapine to aripiprazole, a 61-year-old female with Huntington's disease developed psychosis that persisted despite olanzapine reinstitution. Subsequent hospitalization led to significant complications, including medication adverse effects, infections (parotitis, endocarditis, cystitis), trauma in the setting of restraints, and malnutrition. This case illustrates the risk of medical and pharmacologic complications during prolonged hospitalization, diagnostic challenges in advanced HD, and the importance of multidisciplinary care with early palliative involvement.

Huntington's disease (HD) is a progressive neurodegenerative disorder causing movement, cognitive, and psychiatric symptoms.^1^ Psychiatric manifestations occur in 33–76% of HD patients, with psychotic symptoms in 3–11%.^2–4^ Managing HD is challenging, as treatments targeting one domain may exacerbate others.^5^ With no disease-modifying therapies available and a disease course of 15–20 years, early palliative care integration is considered essential.^1^ We present a case illustrating how inadequate evaluation prior to a medication change may create a preventable cascade of complications in advanced HD.

A 61-year-old female with HD presented with paranoid delusions three weeks after her olanzapine (20 mg daily for approximately seven years) was switched, due to sedation, to aripiprazole (15 mg daily) without cross-titration. She was diagnosed with HD 10 years prior to presentation, with gait abnormalities noted at the time of diagnosis. Her psychiatric history included a prior psychotic episode seven years before this admission, following the deaths of her husband and son, for which she was stabilized on olanzapine 20 mg during an inpatient stay. At the time of this hospital presentation, her mental status examination revealed orientation to person only, significant deficits in both recent and remote memory, concrete thinking, impaired insight and judgment, and bilateral upper extremity chorea. Her pre-admission functional status indicated she had been largely independent in activities of daily living, though her daughter had assumed responsibility for finances and medical appointments — a profile most consistent with HD-ISS Stage 3, defined by measurable impact on daily functioning.^6,7^ Formal HD-ISS assessment tools were not administered; CAG repeat size was unavailable from clinical records, limiting precise disease staging.

Despite olanzapine reinitiation one month later, she failed to return to baseline and, upon admission, exhibited withdrawal, mutism, and stupor concerning for catatonia, though lorazepam challenge (2 mg IV) showed no improvement. After medication adjustments with minimal benefit, refractory agitation required dexmedetomidine, haloperidol, and midazolam, necessitating Medical Intensive Care Unit (MICU) transfer with restraints. Workup, including MRI, EEG, lumbar puncture, and infectious panels, was unremarkable except for generalized background slowing on EEG. Following psychiatry consultation, multiple medication trials were sequentially discontinued or tapered due to adverse effects or intolerance: risperidone (concern for worsening catatonia), valproic acid (thrombocytopenia after three days), and clozapine (oversedation). Concurrently, she developed cascading medical complications: parotitis, atrial fibrillation, a scapular fracture in the context of restraint use, malnutrition requiring feeding tube, and septic shock from a PICC line-associated infection (*Staphylococcus epidermidis* endocarditis, *Klebsiella* and *E. coli* cystitis) requiring daptomycin. Her mental status never fully returned to her premorbid baseline. Nearly four months after initial presentation, she had limited verbal communication, was refusing oral medications, and required total parenteral nutrition and IV antibiotics at home. At the family's request, she was discharged with an outpatient palliative care referral. (Figure 1).

**Figure 1.**
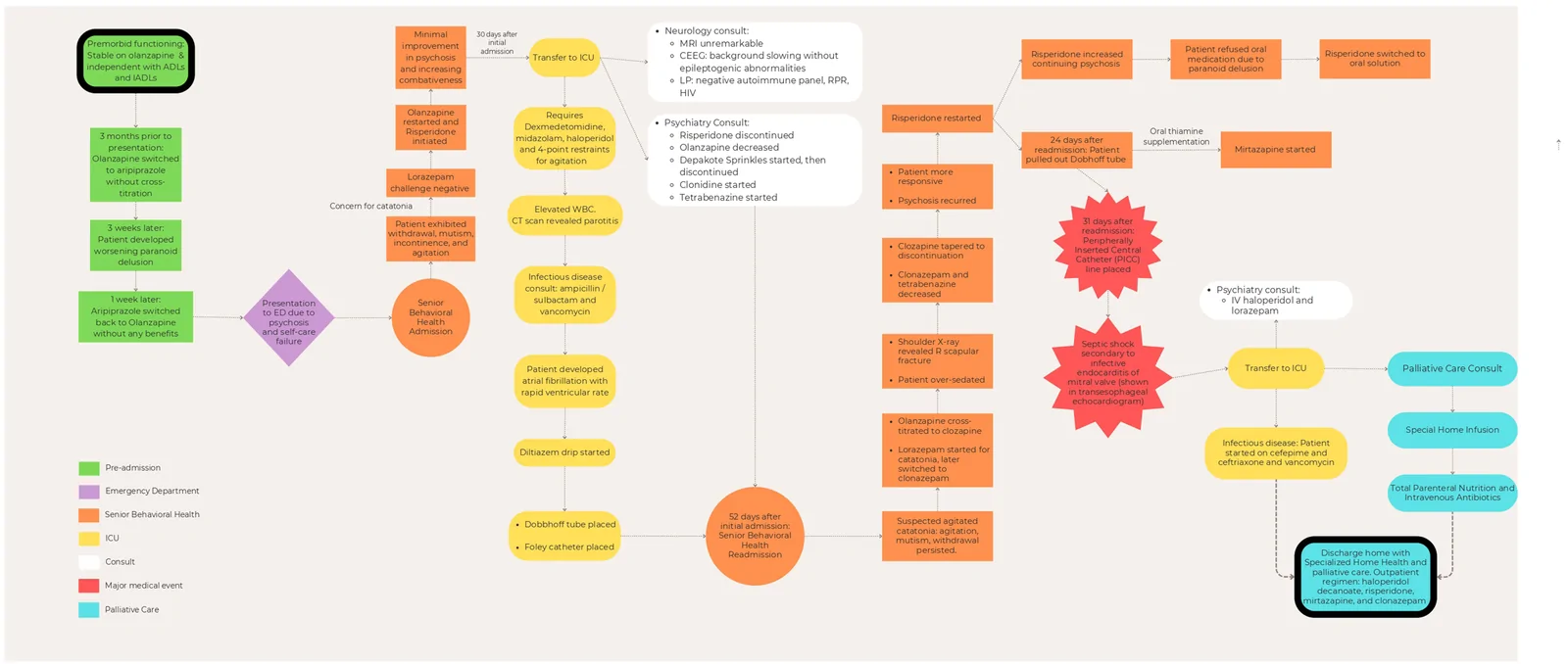
Clinical case summary flowchart. Neuropsychiatric trajectory from olanzapine-to-aripiprazole switch through medical complications to discharge with palliative care.

When a stable HD patient develops subacute deterioration, a broad differential diagnosis should be considered before attributing symptoms to any single cause. Possible contributing factors may have included: (1) disease progression altering antipsychotic sensitivity over time; (2) medical comorbidity, as infection, metabolic derangement, or nutritional deficiency can cause encephalopathy in a cognitively vulnerable patient; (3) medication-related factors, including drug accumulation or interaction (notably with co-prescribed citalopram); and (4) mood disorder, as depression and apathy are common in mid-stage HD and may resemble sedation.^2,9^

It is possible the abrupt transition from olanzapine to aripiprazole may have possibly contributed to psychosis through the dopamine supersensitivity mechanism — one theoretical explanation for the temporal association between the medication switch and symptom onset, though definitive causal attribution is not possible in this retrospective analysis.^8^ Clinicians should consider gradual cross-titration when switching antipsychotics in HD, consistent with expert guidelines.^5^

Distinguishing psychosis, catatonia, delirium, and HD progression proved challenging, given overlapping features.^9^ While catatonia was considered, assessment was limited by underlying chorea and a negative lorazepam challenge. Multifactorial delirium superimposed on HD-associated major neurocognitive disorder emerged as the most plausible working diagnosis, in the setting of multiple concurrent stressors including antipsychotic transition, recurrent infection, malnutrition, and polypharmacy. Patients with HD who have established cognitive impairment may carry a substantially elevated delirium risk, regardless of clinical setting.^10^ Her failure to return to premorbid baseline suggests the acute episode may have contributed to accelerated functional decline.^10^

Polypharmacy contributed to thrombocytopenia^11^ and QTc prolongation risk, prompting citalopram discontinuation. Invasive devices introduced infection risks. This case highlights the risks of aggressive nutritional interventions in advanced neurodegenerative disease; feeding tube placement in neurocognitive decline increases complications,^12^ particularly when patients cannot cooperate with medical devices. Repeated self-removal of the feeding tube necessitated a PICC line, which led to bacteremia and infective endocarditis, a complication with a clear and direct causal link to the catheter. The family's initial reluctance to palliative care involvement reflects the emotional difficulty of transitioning from curative to comfort-focused care.^13^ HD advocacy communities emphasize early palliative involvement,^14^ as it provides caregiver support and aligns treatment with patient goals.

This case demonstrates how neuropsychiatric symptoms can rapidly escalate in HD. Key lessons include: (1) carefully evaluate the reason for any antipsychotic change before switching, and use gradual cross-titration when a transition is indicated; (2) maintain a low threshold for delirium evaluation in HD patients with cognitive impairment, in both hospital and community settings; (3) minimize invasive interventions to reduce multisystem complication risks; (4) engage palliative care early, ideally while patients retain decision-making capacity, to align care with patient and family values before a crisis occurs. Proactive multidisciplinary approaches and transparent family communication are essential to align care with patient values.
